# Synergistic antitumor activity of the combination of salubrinal and rapamycin against human cholangiocarcinoma cells

**DOI:** 10.18632/oncotarget.13408

**Published:** 2016-11-16

**Authors:** Xiaofang Zhao, Chunyan Zhang, Hong Zhou, Bin Xiao, Ying Cheng, Jinju Wang, Fuli Yao, Chunyan Duan, Run Chen, Youping Liu, Chunhong Feng, Hong Li, Jing Li, Rongyang Dai

**Affiliations:** ^1^ Department of Biochemistry and Molecular Biology, Southwest Medical University, Luzhou, Sichuan, China; ^2^ Department of Hepatobiliary Surgery of the Affiliated Hospital, Southwest Medical University, Luzhou, Sichuan, China; ^3^ Department of Public Health, Southwest Medical University, Luzhou, Sichuan, China

**Keywords:** salubrinal, rapamycin, cholangiocarcinoma, Akt, Bcl-xL

## Abstract

Less is known about the roles of eukaryotic initiation factor alpha (eIF2α) in cholangiocarcinoma (CCA). Here, we report that eIF2α inhibitor salubrinal inhibits the proliferation of human CCA cells. Clinical application of mammalian target of rapamycin (mTOR) inhibitors only has moderate antitumor efficacy. Therefore, combination approaches may be required for effective clinical use of mTOR inhibitors. Here, we investigated the efficacy of the combination of salubrinal and rapamycin in the treatment of CCA. Our data demonstrate a synergistic antitumor effect of the combination of salubrinal and rapamycin against CCA cells. Rapamycin significantly inhibits the proliferation of CCA cells. However, rapamycin initiates a negative feedback activation of Akt. Inhibition of Akt by salubrinal potentiates the efficacy of rapamycin both *in vitro* and *in vivo*. Additionally, rapamycin treatment results in the up-regulation of Bcl-xL in a xenograft mouse model. It is notable that salubrinal inhibits rapamycin-induced Bcl-xL up-regulation *in vivo*. Taken together, our data suggest that salubrinal and rapamycin combination might be a new and effective strategy for the treatment of CCA.

## INTRODUCTION

Cholangiocarcinoma (CCA) is highly aggressive tumor arising from the epithelium of intrahepatic and extrahepatic biliary ducts [1–3]. The overall prognosis of CCA is very poor [2]. Resection treatment is the best option for CCA patients. However, resection treatment of CCA is associated with high rates of recurrence, and short survival times [4].

Adjuvant strategies, including chemotherapy, radiotherapy or combination treatment, are used to improve outcomes of CCA patients [5]. Cisplatin, 5-fluorouracil, epirubicin, leucovorin, mitomycin-C and gemcitabine either alone or in combination have been used to treat CCA [6, 7]. The first line chemotherapy for advanced CCA is the combination of gemcitabine and cisplatin [1, 8, 9]. The major drawbacks in clinical applications of cisplatin are the development of resistance by tumors and adverse side effect [7, 10, 11]. Despite intensified evaluation of other chemotherapy combinations with fluorouracil, oxaliplatin or irinotecan, the improvement in survival of CCA patients has been marginal [1, 12]. Several clinical studies have assessed the antitumor activity of molecular targeted therapies in CCA [13–16].

Mammalian target of rapamycin (mTOR) is a critical regulator of cell survival, metabolism, motility, and angiogenesis [17, 18]. Recent evidence suggests that deregulated activation of mTOR is important in CCA [18–22]. Therefore, CCA patients might benefit from mTOR pathway targeted therapies. Although mTOR inhibitors, such as rapamycin and its analogs, are considered as a potential strategy for several cancers, objective response rates with mTOR inhibitors in clinical trials are modest and variable [23, 24]. Recently, a report showed that dual inhibition of PI3K and mTOR by NVP-BEZ235 inhibits CCA cell growth efficiently [20]. It is important to investigate whether combination approaches can enhance the antitumor activity of mTOR inhibitors.

We and others have previously demonstrated that salubrinal, a selective inhibitor of eukaryotic initiation factor alpha (eIF2α), could attenuate or promote the antitumor activities of different chemotherapy agents, such as cisplatin and doxorubicin [25–27]. Whether salubrinal can affect the antitumor activities of mTOR inhibitors remains unknown. We had previously demonstrated that the eIF2α pathway is deregulated in human CCA cells [28], but the antitumor effects of salubrinal in CCA remain to be clarified. In this study, we firstly tried to explore whether salubrinal demonstrates a potential antitumor activity against human CCA cells. Then, we studied the antitumor potential of combined treatment with salubrinal and rapamycin on human CCA cells, and to search potential efficient comprehensive therapeutics for CCA.

## RESULTS

### Rapamycin inhibits the proliferation of CCA cells

**Figure 1:**
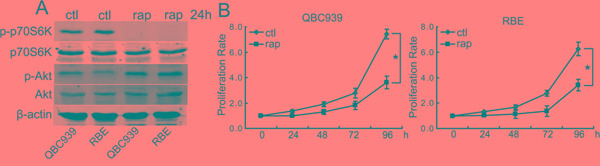
Rapamycin inhibits the proliferation of human CCA cells **A.** Rapamycin inhibits mTOR activity and initiates a negative feedback activation of Akt. After treated with rapamycin (rap, 100 nM) for 24 h, p-p70S6K and p-Akt in QBC939 and RBE cells were analyzed using western blot. **B.** mTOR blocking inhibits CCA cells proliferation. QBC939 and RBE cells were treated with rapamycin (rap, 100 nM) for indicated time periods, and cell viability was determined by CCK8 assay (*P < 0.05).

To determine the roles of mTOR signaling in CCA cells, we firstly studied the effects of rapamycin treatment on the expression of mTOR substrate phosphorylated p70S6K in CCA cells. The data demonstrated that rapamycin efficiently inhibited the phosphorylation of p70S6K in QBC939 and RBE cells (Figure 1A), indicating that rapamycin can inhibit mTOR signaling in CCA cells. It is notable that rapamycin treatment induced phosphorylated Akt increasing in QBC939 and RBE cells (Figure 1A). To determine the anti-proliferation effect of rapamycin on CCA cells, we treated QBC939 and RBE cells with rapamycin for indicated time periods. Compared with dimethyl sulfoxide treatment, rapamycin inhibited the proliferation of QBC939 and RBE cells in a time-dependent manner (Figure 1B). Rapamycin treatment for a relatively long time (48 h) didn’t result in detectable apoptosis of CCA cells (data not shown). These data indicate that aberrant activation of mTOR signaling plays an important role in promoting the proliferation of CCA cells.

### Salubrinal inhibits the proliferation of CCA cells *in vitro* and *in vivo*

**Figure 2:**
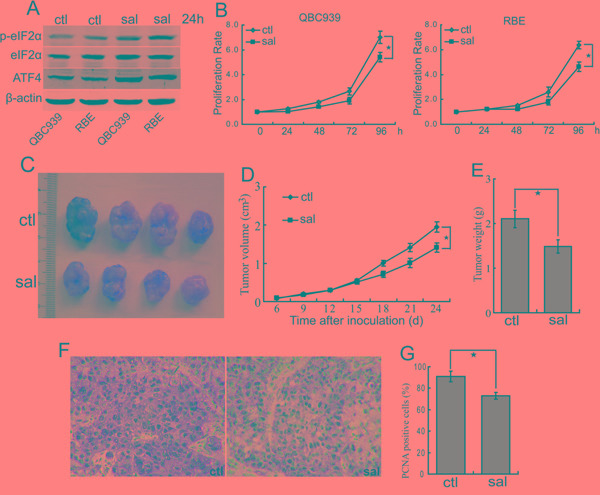
Salubrinal inhibits the proliferation of human CCA cells *in vitro* and *in vivo* **A.** Salubrinal promotes the phosphorylation of eIF2α and expression of ATF4. After treated with salubrinal (sal, 25μM) for 24 h, p-eIF2α and ATF4 in QBC939 and RBE cells were analyzed using western blot. **B.** eIF2α blocking inhibits human CCA cells proliferation *in vitro*. QBC939 and RBE cells were treated with salubrinal (sal, 25μM) for indicated time periods, and cell viability was determined by CCK8 assay (*P < 0.05). **C, D, E.** Salubrinal suppresses the growth of CCA cells in mice. After mice were subcutaneously inoculated with 1×10^7^ QBC939 cells for seven days, mice in the experimental group were administrated with salubrinal (sal, 1 mg/kg i.p, daily). Representative subcutaneous tumors are shown (C). Tumor size was measured every three days from day 6 through 24 after inoculation QBC939 cells into mice (D, *P < 0.05). The final tumor weight of each group was measured (E, *P < 0.05). **F, G.** Salubrinal inhibits CCA cells proliferation *in vivo*. Immunohistochemical staining of PCNA in tumor sections (F) were quantified by counting positive cells in 10 high-power fields (G, *P < 0.05).

We previously demonstrated the eIF2α pathway is deregulated in CCA cells [30]. We set out to investigate the roles of eIF2α signaling in CCA. We examined the expression of phosphorylated eIF2α and its downstream target activating transcription factor 4 (ATF4) in human CCA cells. As shown in Figure 2A, QBC939 and RBE cells showed strong expression of phosphorylated eIF2α and ATF4. Salubrinal treatment, a selective inhibitor of eIF2α, promoted the phosphorylation of eIF2α and the protein levels of ATF4 in QBC939 and RBE cells (Figure 2A), indicating that salubrinal can inhibit the activity of eIF2α in human CCA cells. In order to determine the effects of eIF2α signaling on the proliferation of CCA cells, QBC939 and RBE cells were treated with salubrinal for indicated time periods. The data showed that salubrinal inhibited the proliferation of QBC939 and RBE cells in a time-dependent manner (Figure 2B). It is notable that salubrinal didn’t induce apparent death of QBC939 and RBE cells (data not shown). To further confirm our observation regarding salubrinal on CCA cells, we investigated the effect of salubrinal in inhibition of CCA formation *in vivo*. Salubrinal treatment reduced tumor burden of QBC939 cells (Figure 2C, 2D and 2E). Importantly, salubrinal treatment inhibited the proliferation of QBC939 cells *in vivo* (Figure 2F, 2G). Consistent with our *in vitro* data, salubrinal didn’t induce QBC939 cells apoptosis *in vivo* (data not shown). These data demonstrated that salubrinal suppresses CCA cells growth both *in vitro* and *in vivo*, which imply a potential use of salubrinal in repressing the progression of CCA.

### The combination of salubrinal and rapamycin inhibits CCA xenografts growth *in vivo*

**Figure 3:**
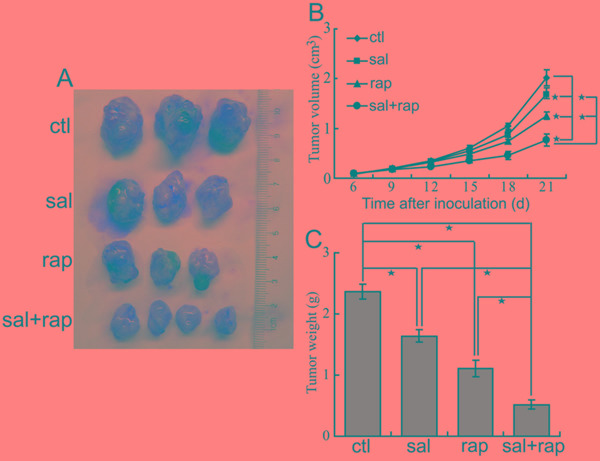
The combination of salubrinal and rapamycin inhibits the growth of CCA xenografts in nude mice **A, B, C.** The combination of salubrinal and rapamycin suppresses growth of CCA xenografts in nude mice. After mice were subcutaneously inoculated with 1×10^7^ QBC939 cells for seven days, mice in the experimental groups were administrated with salubrinal (1 mg/kg i.p, daily), rapamycin (2 mg/kg i.p, daily), salubrinal (1 mg/kg i.p, daily) plus rapamycin (2 mg/kg i.p, daily) respectively. Representative subcutaneous tumors are shown (A). Tumor size was measured every three days from day 6 through 21 after inoculation QBC939 cells into mice (B*, *P* < 0.05). The final tumor weight of each group was measured (C, *P < 0.05).

Consequently, we investigated antitumor activity of combination treatment with salubrinal and rapamycin in nude mice suffering QBC939 CCA xenografts. The results demonstrated that rapamycin treatment significantly inhibited the growth of tumors (Figure 3A, 3B and 3C). Moreover, administration of rapamycin combined with salubrinal displayed more growth inhibition effect compared with rapamycin or salubrinal alone (Figure 3A, 3B and 3C), indicating an additional antitumor effect of the combined treatment. There were not obvious effects on the body weight of mice in animal studies described above (data not shown), indicating that single-agent rapamycin and rapamycin combined with salubrinal are likely well-tolerated.

### The combination of salubrinal and rapamycin inhibits CCA cells growth *in vitro* and *in vivo*

**Figure 4:**
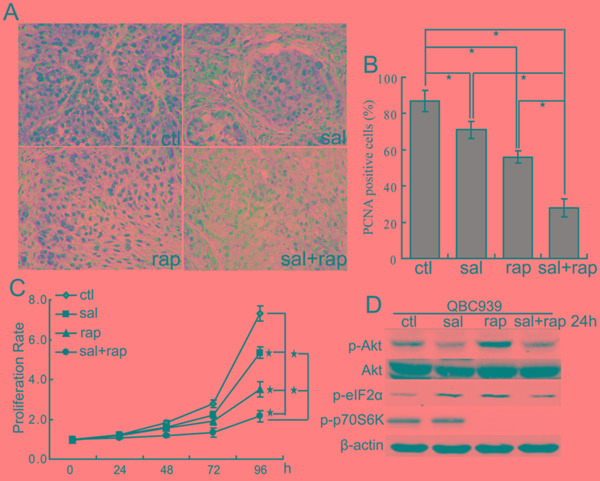
The combination of salubrinal and rapamycin inhibits the growth of human CCA cells *in vitro* and *in vivo* **A, B.** The combination of salubrinal and rapamycin inhibits human CCA cells proliferation *in vivo*. Immunohistochemical staining of PCNA in tumor sections (A) were quantified by counting positive cells in 10 high-power fields (B, *P < 0.05). **C.** The combination of salubrinal and rapamycin inhibits CCA cells proliferation *in vitro*. QBC939 cells were treated with salubrinal (sal, 25μM), rapamycin (rap, 100 nM), salubrinal (sal, 25μM) plus rapamycin (rap, 100 nM) for indicated time periods. Cell viability was determined by CCK8 assay (*P < 0.05). **D.** Salubrinal inhibits the activation of Akt induced by rapamycin treatment. After treated with salubrinal (sal, 25μM) or/and rapamycin (rap, 100 nM) for 24 h, p-Akt, p-eIF2α and p-p70S6K in QBC939 cells were analyzed using western blot.

Since both rapamycin and salubrinal can inhibit the proliferation of CCA cells, we examined the anti-proliferation activity of salubrinal combined with rapamycin. The data showed that combination treatment with salubrinal and rapamycin displayed a significantly higher anti-proliferation activity than treatment with rapamycin or salubrinal alone in nude mice suffering QBC939 CCA xenografts (Figure 4A, 4B). Moreover, the combination of salubrinal and rapamycin induced additional anti-proliferation activity in QBC939 cells *in vitro* (Figure 4C). These results showed that salubrinal and rapamycin work together to suppress CCA cells proliferation *in vivo* and *in vitro*.

To clarify the possible mechanisms underlying the synergistic effect between salubrinal and rapamycin, we checked p-Akt levels after treating cells with salubrinal or/and rapamycin. The results showed that salubrinal treatment not only decreased p-Akt levels but also inhibited rapamycin-mediated the increase of p-Akt levels in QBC939 cells (Figure 4D). These findings indicate that salubrinal and rapamycin might exert the synergistic effects, at least in part, through regulating Akt signaling.

### The combination of salubrinal and rapamycin induces apoptosis of CCA cells *in vivo*

**Figure 5:**
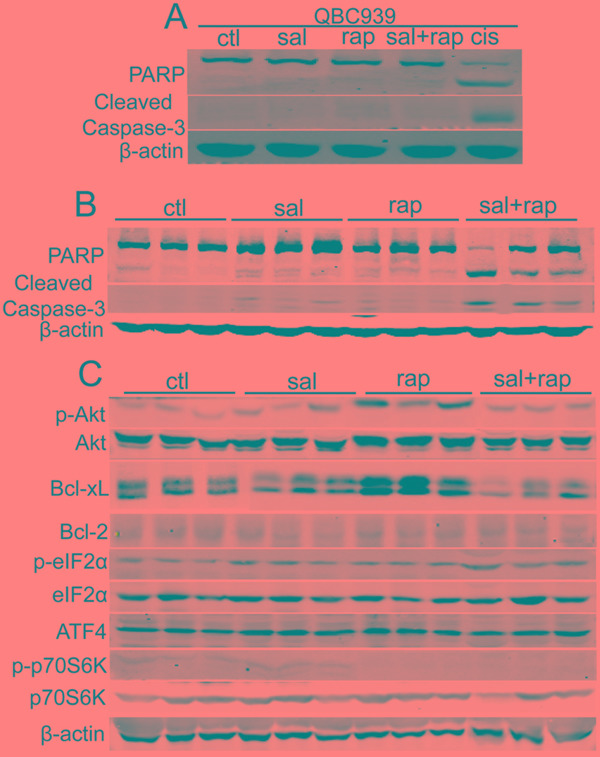
The combination of salubrinal and rapamycin induces apoptosis of human CCA cells *in vivo* **A.** The combination of salubrinal and rapamycin does not induce CCA cells apoptosis *in vitro.* After treated with salubrinal (sal, 25μM) or/and rapamycin (rap, 100 nM) for 48 h, PARP and cleaved caspase-3 in QBC939 cells were analyzed using western blot. Cisplatin (cis, 10 μg/mL, 24 hour) treatment was used as positive control. **B.** The combination of salubrinal and rapamycin augments apoptosis of QBC939 cells *in vivo*. PARP and cleaved caspase-3 in tumor tissues (from Figure 3A) were analyzed using western blot. **C.** Salubrinal inhibits rapamycin-induced Bcl-xL and p-Akt up-regulation of QBC939 cells *in vivo*. p-Akt, Bcl-xL and Bcl-2 in tumor tissues (from Figure 3A) were analyzed using western blot.

As salubrinal can inhibit rapamycin-induced Akt activation in CCA cells, we investigated whether the combination of salubrinal and rapamycin can initiate the apoptosis of CCA cells. The results demonstrated that salubrinal and rapamycin alone or combination didn’t initiate the apoptosis of QBC939 and RBE cells *in vitro* (Figure 5A). Moreover, salubrinal or rapamycin alone didn’t induce apparent cleavage of PARP or caspase-3 of QBC939 cells *in vivo* (Figure 5B). It is notable that salubrinal and rapamycin combination induced apparent cleavage of PARP and caspase-3 of QBC939 cells *in vivo* (Figure 5B). Thus, the combination of salubrinal and rapamycin induces apoptosis of QBC939 cells *in vivo*.

To clarify the possible mechanisms underlying the synergistic effect between salubrinal and rapamycin in apoptosis induction *in vivo*, we checked p-Akt, Bcl-2 and Bcl-xL levels after cells were treated with salubrinal or/and rapamycin. Consistent with our *in vitro* data, rapamycin-induced p-Akt increasing was inhibited by salubrinal *in vivo* (Figure 5C). However, salubrinal treatment had no apparent effects on p-Akt levels *in vivo* (Figure 5C). Importantly, rapamycin treatment increased the levels of Bcl-xL of QBC939 cells *in vivo*, and this increasing was repressed by salubrinal (Figure 5C). Moreover, no apparent changes of Bcl-2 protein levels were observed upon salubrinal or/and rapamycin treatment (Figure 5C). Together, these findings indicate that salubrinal and rapamycin combination-mediated p-Akt and Bcl-xL decreasing is involved, at least in part, in the synergistic apoptosis effects of these two agents in QBC939 cells *in vivo*.

### The combination of ABT-737 and rapamycin inhibits the growth of CCA xenografts in nude mice

**Figure 6:**
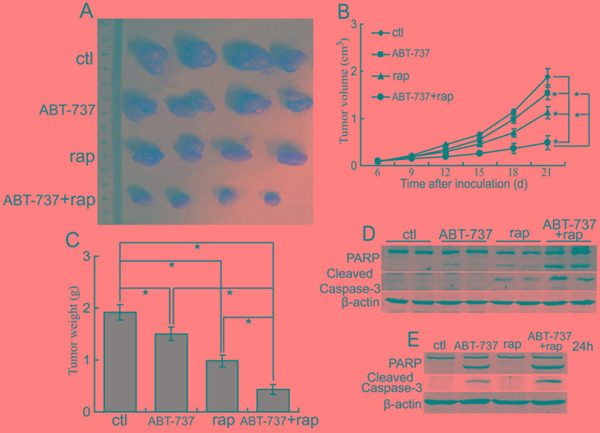
ABT-737 augments the antitumor effect of rapamycin **A, B, C.** The combination of ABT-737 and rapamycin suppresses growth of CCA xenografts in nude mice. After mice were subcutaneously inoculated with 1×10^7^ QBC939 cells for seven days, the experimental groups mice were treated with ABT-737 (50 mg/kg i.p, daily), rapamycin (2 mg/kg i.p, daily), ABT-737 (50 mg/kg i.p, daily) plus rapamycin (2 mg/kg i.p, daily) respectively. Representative subcutaneous tumors are shown (A). Tumor size was measured every three days from day 6 through 21 after inoculation QBC939 cells into mice (B, *P < 0.05). The final tumor weight of each group was measured (C, *P < 0.05). **D.** The combination of ABT-737 and rapamycin augments apoptosis of QBC939 cells *in vivo*. PARP and cleaved caspase-3 in tumor tissues (from A) were analyzed using western blot. **E.** The combination of ABT-737 and rapamycin augments apoptosis of QBC939 cells *in vitro*. After treated with ABT-737 (5μM) or/and rapamycin (rap, 100 nM) for 24 h, PARP and cleaved caspase-3 were analyzed using western blot.

To investigate whether Bcl-xL up-regulation inhibits the antitumor activity of rapamycinin CCA, we treated nude mice suffering QBC939 xenografts with Bcl-xL inhibitor ABT-737 or/and rapamycin. The results demonstrated that ABT-737 treatment inhibited the growth of tumors (Figure 6A, 6B and 6C). Moreover, administration of ABT-737 combined with rapamycin displayed more antitumor effects compared with ABT-737 or rapamycin alone (Figure 6A, 6B and 6C). There were not obvious effects on the body weight of mice in animal studies described above (data not shown), indicating that single-agent ABT-737 and ABT-737 combined with rapamycin are likely well-tolerated. These data indicate that the combination treatment exerts additional antitumor activity.

Considering that Bcl-xL is an important antiapoptosis molecule, we investigated whether rapamycin combined with ABT-737 can initiate the apoptosis of CCA cells. The results showed that ABT-737 and rapamycin combination induced more obvious cleavage of PARP and caspase-3 of QBC939 cells *in vivo* (Figure 6D). Moreover, ABT-737 and rapamycin combination induced the cleavage of PARP and caspase-3 of QBC939 cells *in vitro* (Figure 6E). Thus, salubrinal augments the antitumor effect of rapamycin, at least in part, through inhibiting the expression of Bcl-xL.

## DISCUSSION

The mTOR pathway, which is often hyperactivated in cancer cells, is implicated in the carcinogenesis and progression of CCA [28–31]. This pathway can promote cancer cell growth as well as migration and invasion [30, 32]. It is known that inhibiting the proliferation and invasion of malignant biliary epithelial cells is a potential strategy for the treatment of CCA. Thus, mTOR inhibition might be a promising strategy for the treatment of CCA. Emerging data indicate that mTOR inhibitors only had moderate antitumor efficacy [33–35]. There are reports indicate that several molecular mechanisms, such as Akt activation induced by mTOR inhibition, might account for the limited antitumor effects of mTOR inhibitors [35–37].

In this study, we firstly analyzed the antitumor effects of mTOR inhibitor rapamycin in human CCA cells. Our data are consistent with the observation that rapamycin effectively inhibited the growth of CCA cells [35]. We also found that rapamycin treatment resulted in the activation of Akt in QBC939 and RBE cells. It is notable that rapamycin treatment induced the increase of p-Akt in a subcutaneous xenograft model. Since Akt signaling plays a key role in cancer cell proliferation and antiapoptosis, it is reasonable to suggest that mTOR-Akt negative feedback might play an essential role in suppressing the antitumor efficacy of rapamycin in CCA. It has been reported that combined targeting of Akt and mTOR can suppress CCA effectively [35]. Thus, combined inhibition of Akt and mTOR might be a potential therapeutic approach for CCA patients.

We and others have previously reported that the sensitivity of cancer cells to cisplatin and doxorubicin can be changed by eIF2α specific inhibitor salubrinal [25, 26, 38]. Here, our data show that salubrinal treatment inhibited the proliferation of human CCA cells *in vitro* and *in vivo*. Importantly, salubrinal combined with rapamycin synergistically inhibited the proliferation of CCA cells. Moreover, the combination of salubrinal and rapamycin induced CCA cells apoptosis *in vivo*, but not *in vitro*. Interestingly, our *in vitro* and *in vivo* data demonstrated that rapamycin-mediated p-Akt up-regulation was blocked by salubrinal in CCA cells. These results strongly suggest that salubrinal can augment the antitumor efficiency of rapamycin through Akt targeting.

An important question now before us is how combined salubrinal and rapamycin promoted apoptosis of CCA cells *in vivo*, but not *in vitro*. In addition to Akt, salubrinal might augment the antitumor effect of rapamycin in CCA cells through other cellular mechanisms. This speculation is supported by our *in vivo* data which demonstrated that rapamycin induced the up-regulation of Bcl-xL, and this up-regulation was inhibited by salubrinal treatment. To further confirm the roles of the combination of salubrinal and rapamycin in Bcl-xL regulation, we investigated whether salubrinal can inhibit Bcl-xL protein levels in CCA cells upon rapamycin treatment *in vitro*. Our data showed that neither salubrinal nor rapamycin had apparent affect on the protein levels of Bcl-xL in CCA cells *in vitro* (data not shown). This difference is likely due to the differences between *in vitro* and *in vivo* environment. The *in vivo* tumor cells suffer from a complex environment lacking nutrient and oxygen supplement with various stress, such as oxidative stress and endoplasmic reticulum stress. The tumor cells cultured *in vitro* are usually in more favorable environment. Thus, it is rational that combined salubrinal and rapamycin regulates Bcl-xL and triggers cell apoptosis *in vivo* but not *in vitro*. In our opinion, the *in vivo* results may recapitulate the clinical response more faithfully. To determine the roles of Bcl-xL in protecting CAA cells against rapamycin treatment, we investigated the effects of Bcl-xL inhibition on the antitumor effects of rapamycin in CCA cells. Based on our findings, we proposed that the combination of Bcl-xL inhibitor ABT-737 and rapamycin synergistically inhibits the growth of CCA cells *in vivo*. In addition, the combination of ABT-737 and rapamycin promoted CCA cells apoptosis. Taken together, these data may explain, in part, the differential cell killing response of the combination of salubrinal and rapamycin of CCA cells between *in vitro* and *in vivo*.

In conclusion, salubrinal exerts direct antitumor effects on CCA through suppressing the growth of CCA cells, and the combination of salubrinal with rapamycin demonstrated a synergistic antitumor effect by regulating Akt and Bcl-xL signaling. These findings shed light on the molecular basis of eIF2α inhibitors alone or in combination with mTOR inhibitors and a possible new strategy for the treatment of CCA.

## MATERIALS AND METHODS

### Chemicals and antibodies

Cell counting kit-8 (CCK8) was purchased from Sigma (Lyon, France). eIF2α phosphatase enzymes inhibitor salubrinal and mTOR inhibitor rapamycin were purchased from Tocris Bioscience (Bristol, UK). Bcl-xL inhibitor ABT-737 was purchased from Selleck Chemicals (Houston, TX, USA). Antibodies against p-p70S6K, p70S6K, p-Akt (S473), Akt, p-eIF2α (S51), eIF2α, PCNA, PARP, Cleaved Caspase-3, Bcl-xL and Bcl-2 were purchased from Cell Signaling Technology (Danvers, MA, USA). Antibodies against ATF4 and β-actin were purchased from Santa Cruz Biotechnology (Heidelberg, Germany).

### Cell culture

Human CCA cell lines QBC939 and RBE were cultured in RPMI-1640 medium supplemented with 10% fetal bovine serum and 1% penicillin/streptomycin in a humidified incubator containing 5% CO_2_ and 95% ambient air at 37 °C.

### Western blot

Cells were lysed in Triton lysis buffer (20 mM Tris, pH 7.4, 137 mM NaCl, 10% glycerol, 1% Triton X-100, 2 mM EDTA, 1 mM PMSF, 10 mM NaF, 5 mg/ml aprotinin, 20 mM leupeptin, and 1 mM sodium orthovanadate) and centrifuged at 12,000 g for 15 min. Protein concentrations were measured using the BCA assay. Protein samples were denatured with 4 × SDS-loading buffer (200 mM Tris, pH 6.8, 8% SDS, 400 mM DTT, 0.4% bromphenol blue, 40% glycerol) at 100 °C for 5 min and subjected to standard SDS-PAGE and western blot analysis as previously described [41].

### Cell proliferation assay

QBC939 and RBE cells were trypsinized and seeded at 3 × 10^3^ cells/well in 96-well plates. After 24 h, inhibitors were added and incubated for indicated time periods. Then, 20 μL of CCK8 solution (5 g/L) in phosphate buffered saline (PBS) was added. After incubated for an additional 2 h, the absorbance value in each well was measured using a microculture plate reader (Bio-Tek, USA) at a wavelength of 490 nm.

### Tumor xenograft experiments

The use of animals in present study has been approved by the local committee on animal care. Six-week-old male nude mice were purchased from the Shanghai Experimental Center (CSA, Shanghai, China). Mice were subcutaneously inoculated with QBC939 cells. Approximately 1×10^7^ cells in 0.2 ml culture medium containing phosphate-buffered saline were injected subcutaneously into the right flank of the mice, which were then observed daily for signs of tumor development. Tumor volume was calculated as below: *V* (cm^3^) = width^2^ (cm^2^) × length (cm)/2. Seven days after inoculation, mice were randomly divided into groups (n=6). Intratumoral injection was performed to the experimental group mice with salubrinal (1 mg/kg i.p, daily), rapamycin (2 mg/kg i.p, daily), salubrinal (1 mg/kg i.p, daily) plus rapamycin (2 mg/kg i.p, daily), ABT-737 (50 mg/kg i.p, daily), ABT-737 (50 mg/kg i.p, daily) plus rapamycin (2 mg/kg i.p, daily). The control group mice were treated by intratumoral injection of physiological saline daily. After treatment for indicated time periods, mice were sacrificed and tumors were excised and weighed.

### Immunohistochemistry

Tissue samples embedded in paraffin were cut into 5μm sections and stained according to standard immunohistochemistry protocols.

### Statistical analysis

Results were expressed as the mean ± S.D. Statistical analysis was performed using Student's t test. *p* < 0.05 was considered statistically significant.
